# Alignment of library services with the research lifecycle

**DOI:** 10.5195/jmla.2019.595

**Published:** 2019-07-01

**Authors:** Bart Ragon

**Affiliations:** Associate Director for Knowledge Integration, Research, and Technology, Claude Moore Health Sciences Library, University of Virginia, Charlottesville, VA, bart@virginia.edu

## Abstract

**Objectives:**

This study sought to understand the needs of biomedical researchers related to the research lifecycle and the present state of library support for biomedical research.

**Methods:**

Qualitative interview data were collected from biomedical researchers who were asked to describe their research activities from identifying a problem to measuring the impact of their findings. Health sciences library leaders were surveyed about the services that they currently provide or plan to provide in supporting biomedical research.

**Results:**

Library services were strongest at the beginning and end of the research lifecycle but were weaker in the conducting phase of research. Co-occurrence of codes from the qualitative data suggests that library services are on the fringe of rather than integrated into the research lifecycle.

**Discussion:**

Findings from this study suggest that tradition-based service models of health sciences libraries are insufficient to meet the needs of biomedical researchers. Investments by libraries in services that integrate with the conducting phase of research are needed for libraries to remain relevant in their support of the research lifecycle.

## INTRODUCTION

Health sciences libraries have traditionally acted as the gatekeepers of medical knowledge. The legitimacy of libraries as gatekeepers to biomedical knowledge in academic health sciences centers is being challenged by exogenous shocks in information technologies, which are changing the norms for how medical knowledge is created and accessed. To maintain their relevance in academic health sciences centers, libraries need to adapt quickly to the shifting landscape of biomedical research.

Research lifecycle models have emerged in an attempt to define the research workflow from identifying problems to measuring the impact of published findings. In 2012, the University of Central Florida Libraries developed a research lifecycle model illustrating the steps of the research lifecycle and identifying the services that libraries and other university departments provide [1]. In 2013, Vaughan et al. explored how niche services by individual librarians could be developed into a standard service model supporting the entire research lifecycle [2].

More recently, questions have emerged concerning the role of health sciences libraries in supporting data. In 2015, the National Institutes of Health (NIH) strategic vision called “for NIH to position the [National Library of Medicine] NLM as a unifying force in biomedicine that promotes and accelerates knowledge generation, dissemination and understanding in the United States and internationally” [3]. The vision also calls on NLM to become the epicenter for biomedical data science across the biomedical research enterprise. Some health sciences libraries are investigating ways to become the epicenter for biomedical data at their institutions, and Federer has found that data librarians provide a broad range of services to researchers [4].

Despite interest from libraries in understanding needs related to the research lifecycle or developing data services, questions remain about what roles are appropriate for libraries in supporting biomedical research. To further understand the connection between libraries and biomedical research, this study explored the demands placed on biomedical researchers and the support services provided by health sciences libraries.

## METHODS

### Study design

The study utilized a convergent parallel design, collecting qualitative and quantitative data independently and comparing or relating the results [5]. Qualitative data were collected from biomedical researchers through semi-structured interviews. Quantitative data were collected from health sciences library leaders using a survey instrument. A document analysis of research lifecycle models (supplemental Appendix A) assisted in creating a semi-structured interview instrument, which was designed to create a dialogue with participants to better understand the demands of biomedical research. The survey instrument that was administered to health sciences library leaders was initially informed by the document analysis of research lifecycle models and later by emergent codes identified during the biomedical researcher interviews. Triangulating data assisted in identifying connections and gaps between health sciences library services and needs related to the research lifecycle.

Data from the qualitative interviews and quantitative survey were analyzed using SPSS version 24.0, Dedoose, VOSviewer 1.6.6, and Microsoft Excel 2016. Dedoose, a web-based qualitative and mixed methods tool for analyzing data, assisted in analyzing transcribed audio interview data collected from biomedical researchers. Qualtrics, a web-based survey tool, was used to administer the survey instrument to health sciences library leaders.

Findings from this study can assist health sciences library leaders develop strategies for supporting biomedical research at their institutions. Transferability, rather than generalizability, was the aim of this study. In transferability, the applications of findings into another context is best determined by the one making the transfer rather than the researcher who conducted the study [6]. This study was approved under University of Virginia Institutional Review Board (IRB)-SBS protocol number 2017-0156.

### Sites for data collection

A purposive approach was used to identify biomedical researchers and health sciences library leaders to ensure collection of a diverse pool of data. The Blue Ridge Institute for Medical Research (BRIMR) produces an annual ranking of NIH-funded medical schools in the United States [7]. Use of BRIMR rankings for this study assisted in categorizing potential site locations using NIH funding as a metric to gauge the level of research activity and yielded 139 potential sites. BRIMR data were then cross-referenced with Association of Academic Health Sciences Libraries members to create a unified list of 112 total potential sites for the study (supplemental Appendix B). The potential sites were divided into quintiles, based on funding, so that a diverse sample of participants could be assembled from among different types of institutions.

### Biomedical researcher interviews

Awardees of NIH Research Grants (R series), Career Development Awards (K series), and Research Training and Fellowship (T and F series) awards from the unified list of potential sites were used as criteria for participation in the study. Diversity among quintiles and stage of career was sought to obtain a variety of perspectives from researchers. NIH RePORTER was mined for potential participants, and biomedical researcher email discussion lists were used to solicit participation. Personal and professional contacts, as well as snowball sampling techniques, were used to identify potential participants and solicit participation from biomedical researchers.

Participants were allowed to deviate from the semi-structured questions so that emergent codes could be explored. Analysis of the interview data utilized open coding techniques, which allowed the data to be investigated and conceptual categories to emerge [8]. Each interview was coded within one week of recording, and interview data that had been previously analyzed were recoded to incorporate emergent codes.

In many cases, participants described aspects of their research that yielded multiple codes. When applicable, codes were allowed to co-occur, and this co-occurrence was later used in the analysis of findings using a network analysis tool. At the completion of the analysis process, all interviews were reviewed and recoded as needed to increase reliability of code application throughout the data. Data were collected until saturation of codes occurred. Saturation, which is a widely accepted practice in qualitative research, generally occurs when there are mounting instances of the same codes and no new codes are emerging from the data [9]. Guest et al. refer to saturation as the “gold standard by which purposive sample sizes are determined in health science research” [10].

Qualitative data were collected through in-person, phone, and online interviews (supplemental Appendix C) that were designed to capture researcher practices in biomedical research from idea inception to impact of findings.

### Health sciences library leader survey

Library directors who met the selection criteria were emailed directly to solicit their participation. One member from each site was allowed to complete the survey, as long as they held the position of director, deputy director, associate director, or other leadership role likely to be involved with current or planned services supporting biomedical research.

A survey instrument (supplemental Appendix D) was administered to the leaders of academic health sciences libraries using the selection criteria. Library leaders were asked to complete a survey about existing research support services and plans for new services. Open-ended questions in the survey solicited feedback on respondent’s perception of the libraries’ role in the research lifecycle and the steps needed to meet the evolving needs of researchers.

## RESULTS

### Biomedical researcher interviews

The 17 biomedical researchers who were interviewed for this study worked at 9 different institutions. Seven of the researchers were female, and 10 were male. Established researchers were the most common (n=8), closely followed by trainees (n=7) and then early career researchers (n=2).

The data were analyzed using 47 unique codes that constituted the initial code set and codes that emerged from the data through the use of open coding techniques (supplemental Appendix E). In total, 1,196 codes were applied to the data and organized into 5 conceptual categories: planning research, conducting research, disseminating research, assessing research impact, and general (supplemental Appendix F).

#### Planning research

Researchers were asked to describe their major activities when planning a research project prior to data collection, analysis, and interpretation. Grant preparation, literature searching, methodology, and identification of collaborators emerged as the most frequently coded activities during research planning.

Researchers were also asked to describe their comfort level in searching for and retrieving literature. All researchers expressed a high degree of comfort conducting searches themselves. Some researchers, while first expressing comfort in conducting their own searches, also spoke of the value of working with librarians on complex search needs. In addition, many researchers expressed the importance of library collections and remote access. However, while many of the researchers mentioned using library support for literature searching, a substantial number of researchers did not associate library support with literature searching, and even fewer used library services for assistance with systematic reviews.

Interestingly, attending conferences was predefined as a code under the concept of disseminating research but emerged as an important concept for planning research. Researchers described attending conferences as an important way to identify collaborators. Attending professional conferences allowed researchers to connect with their colleagues and discover new colleagues, and allowed others to discover them.

#### Conducting research

Researchers were asked to describe the major activities required to begin collecting and analyzing data. The most frequent codes pertained to data analysis, collaboration, data collection, and data management. Researchers’ description of data collection, data management, and data analysis were interconnected and not described as distinct activities. As researchers described management of their data, they addressed concepts of storage, privacy, security, and cleaning. No researcher referred to the need for data management plans, such as those required by some funders and often supported by libraries. Several researchers acknowledged that recent trends from federal funding agencies incentivized external collaboration among researchers.

#### Disseminating research

Researchers were asked to describe the major activities that are required to disseminate their research findings. Not surprisingly, publishing in peer-reviewed journals was the primary currency for disseminating their findings. Researchers were not concerned about author rights, with three researchers believing that the library or university purchased the copyright of their publication.

Open access was frequently referenced in the interviews, likely due to the interviewer’s interest in understanding the connection of open access practices as an emergent code in biomedical research. Researchers were aware of open access compliance policies associated with NIH funding. Most references to open access related to NIH policy requirements rather than a general desire to make their published work openly available.

Researchers described using social media as a communication tool to promote their research online. Specific social media tools mentioned included ResearchGate, LinkedIn, Twitter, and Instagram. ResearchGate and LinkedIn were also referenced as ways to manage their professional identities. Twitter and Instagram accounts were described as tools for interacting with other researchers but mainly for promoting their findings or celebrating accolades. Along with individual Twitter and Instagram accounts that researchers maintained, some labs had accounts.

#### Assessing research impact

Researchers were asked to describe the major activities required to assess the impact of their research findings. Most researchers did not state that they used citation metrics as a way to measure the impact of their research. Journal impact factor was used as a measure for journal selection. Publishing in high-impact journals was perceived as a way to generate more exposure to their work, thus creating the potential for more citations. Most researchers described the process of journal selection as finding a field-relevant journal and then choosing the journal with the highest impact number.

Altmetrics were not frequently referenced, although one researcher linked the value of altmetrics to changes in the NIH Biosketch section C, which asks researchers to describe their contributions to science. One researcher relied heavily on support from the library, linking his lab’s successful track record in obtaining funding to library assistance with Biosketches. He described the library’s impact service as “revolutionary” and the “secret sauce” of their grant applications.

#### General codes

Several codes emerged from the data that were not part of the interview probes for planning research, conducting research, disseminating research, and assessing research impact. General category codes were most prominently connected to support mechanisms for research. Researchers identified support coming from a variety of places, but most frequently in the form of administrative support, biostatisticians, and librarians. The relationship between mentors and mentees emerged as the most frequently coded item in the data. Both mentors and mentees relied on this relationship to accomplish their individual goals. Mentors need the mentees to act as the workforce of the lab, and mentees need mentors to help them establish their reputations.

### Research lifecycle visualized through co-occurrence of codes

**Figure 1:**
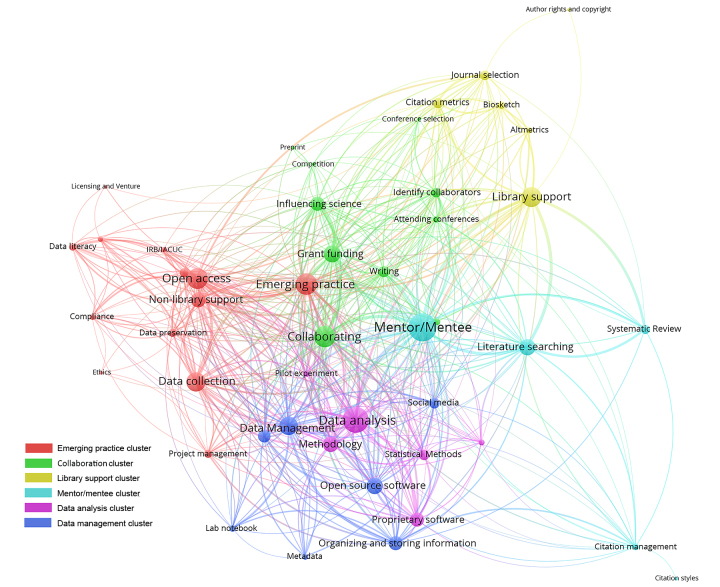
Co-occurrence of codes within the research lifecycle

A graph database was created from co-occurrence data suggesting a relationship between research lifecycle codes. Graph databases help to explore patterns and relationships in data that are hard to distinguish with numeric or string values. VOSviewer was used to create the visualization, which calculates the associated strength between data objects using a similarity measure. Also referred to as probabilistic affinity, similarity measures display how alike two or more data objects are to each other by calculating the strength of their association [11]. All forty-seven co-occurring codes met the VOSviewer threshold for inclusion. The parameter for link strength was set to zero so that all co-occurring codes could be represented in the visualization. Figure 1 shows the relationship of co-occurrence data from the codes, as described by biomedical researchers. The dominant code was used to name the six major clusters identified by the similarity measure: emerging practice, collaboration, library support, mentor/mentee, data analysis, and data management.

The close proximity of activities between the emerging practice, data analysis, and data management clusters illustrates the entanglement between the various processes supporting the research lifecycle. Emerging practice connects with codes such as open access, data collection, data literacy, and compliance. Data analysis is more connected to methodology, statistical methods, and proprietary software. Data management is connected with reproducibility and replicability as well as metadata and organizing and storing information. Many activities in these three clusters represent processes associated with the conducting phase of the research lifecycle.

Collaboration and mentor/mentee clusters refer primarily to activities associated with the planning research phase. Collaboration clustered with concepts like finding grant funding, influencing science, identifying collaborators, and attending conferences. Mentor/mentee was most frequently connected with literature searching, systematic reviews, and citation management. Although the collaboration and mentor/mentee clusters are located in the center of the figure, they connect primarily with activities outside of the high density of activities in the lower left quadrant, suggesting that these activities support the conducting phase of research.

These co-occurrence data reveal a number of interesting relationships considering library support and the research lifecycle. The library support cluster includes journal selection, citation metrics, altmetrics, and biosketch. The library support node is large in size, suggesting a strong support need from researchers. However, the location of the library support node is the furthest from the center of all six clusters, being on the outside border of the visualization and distant from the high density of connections in the center of the visualization.

### Health sciences library leader survey

Forty-six percent of the 112 library leaders participated in the study. The distribution of health sciences library leaders who completed the survey (n=51) included executive director, director, or similar level of responsibility (n=44); deputy or associate director (n=3); and assistant director, department head, or similar level of responsibility (n=4). These library leaders reported that their libraries provided or planned to provide research support services. Survey results were categorized into the four conceptual categories of planning research, conducting research, disseminating research, and assessing research impact.

#### Planning research

**Figure 2:**
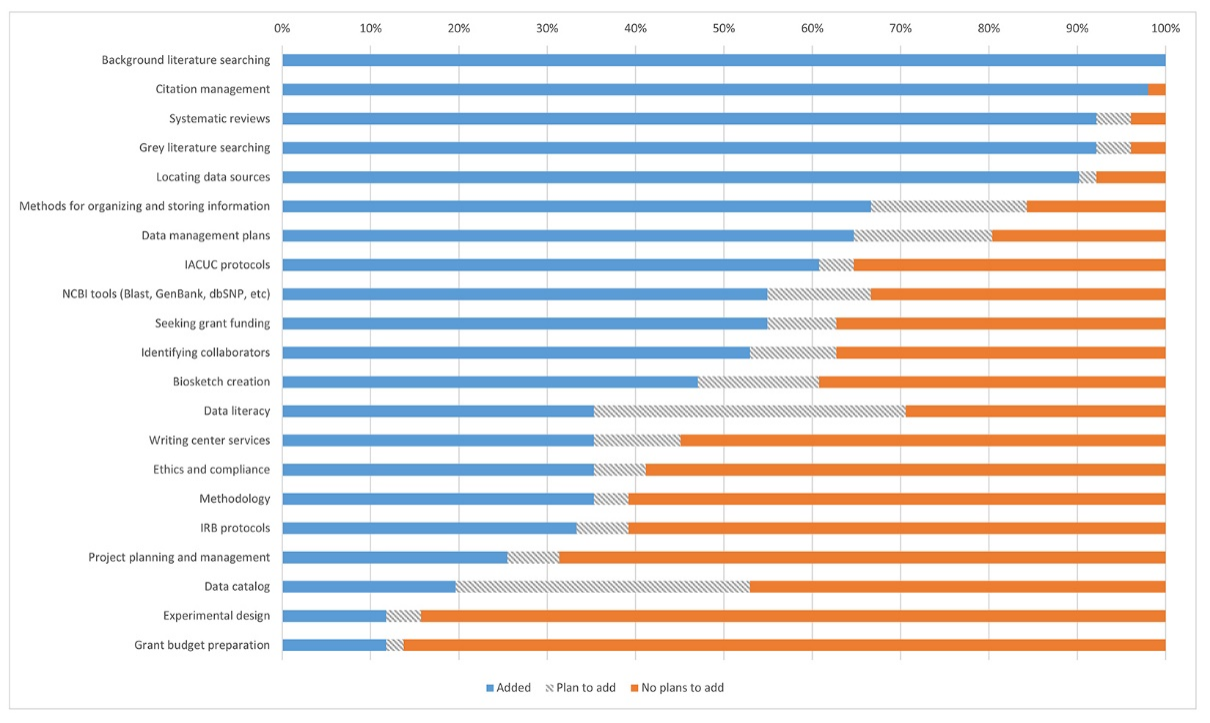
Library support for research planning activities

The survey results indicated a strong presence of library services for research planning support (Figure 2). At least 50% of library leaders indicated that they currently provided support services for background literature searching, citation management, systematic reviews, grey literature, location of data sources, methods for organizing and storing information, data management plans, institutional animal care and use committee protocols, National Center for Biotechnology Information tools, searches for grant funding, and identification of collaborators. Services least likely to be supported by health sciences libraries included writing center services, ethics and compliance, methodology, IRB protocols, project planning and management, experimental design, and grant budget preparation.

Library leaders reported mixed results when it came to data literacy and data catalog services. Thirty-five percent of libraries supported data literacy, with an additional 35% planning to add services. Similarly, 20% of libraries supported data catalogs, with 33% more planning to add this service.

#### Conducting research

**Figure 3:**
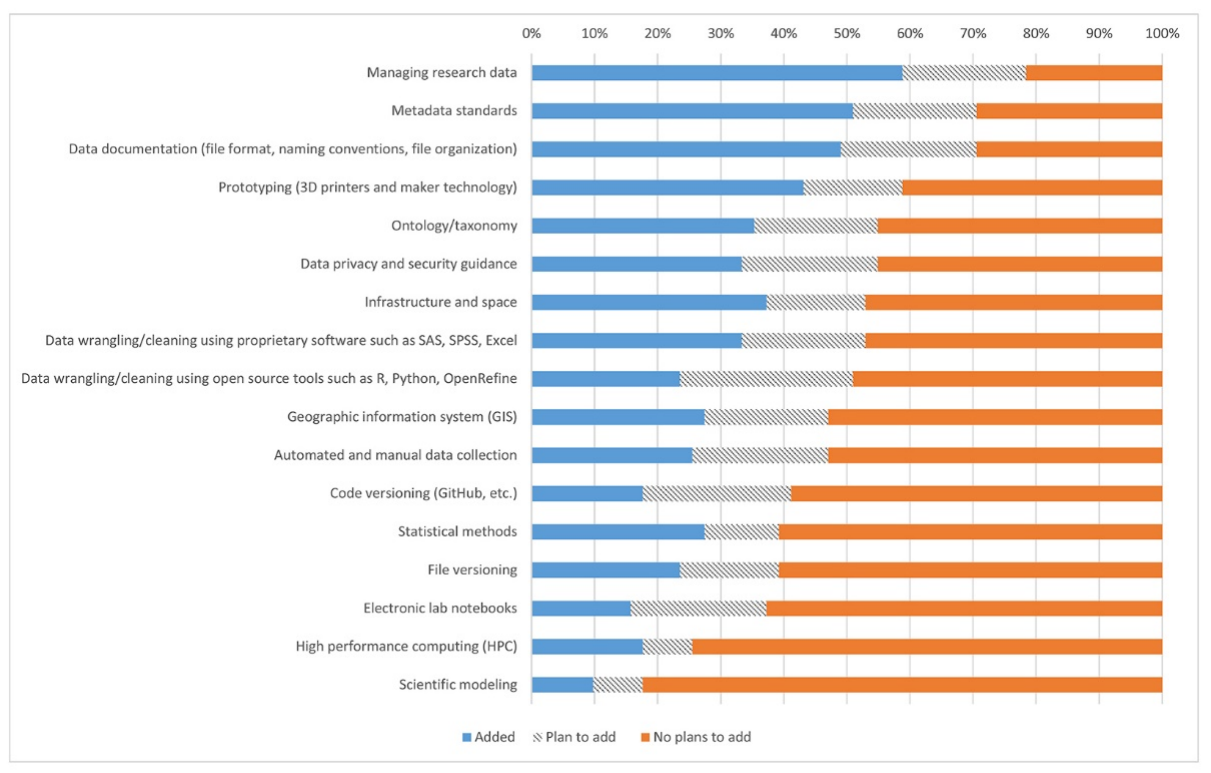
Library support for conducting research activities

Support for managing research data and metadata standards were the only services provided by 50% or more libraries, although many libraries planned to expand their data-related services supporting the conducting phase of research (Figure 3). Forty-nine percent of libraries provided services for data documentation, with 22% more planning to add this service. Thirty-three percent of libraries provided services for data wrangling or cleaning using proprietary software, with an additional 20% planning to add this service. Similarly, 24% of libraries provided services for data wrangling or cleaning using open source tools, with 27% planning to add this service. An additional 20% of libraries were planning to add services related to managing data.

Even though more than 40% of libraries had or planned to add services related to data in the conducting phase, more than 40% did not plan to add services for data wrangling with proprietary or open source software, data privacy and security, or data collection.

#### Disseminating research

**Figure 4:**
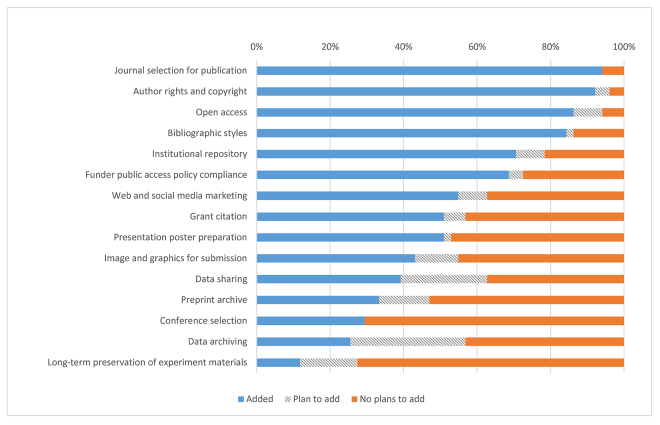
Library support for disseminating research activities

More than half of the library leaders indicated that they provided services for journal selection for publication, author rights and copyright, open access, bibliographic styles, institutional repositories, funder public access policy compliance, web and social media marketing, grant citation, and presentation poster preparation (Figure 4). The majority of library leaders did not indicate that services related to long-term preservation of experiment materials and data archiving were support roles that they were considering for their libraries.

#### Assessing research impact

**Figure 5:**
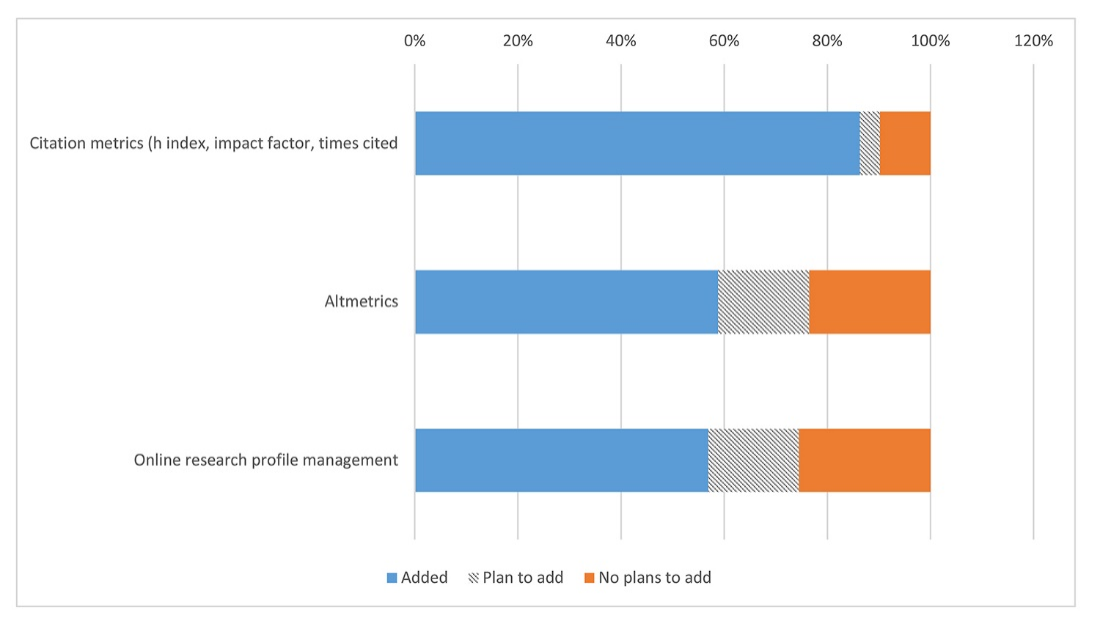
Library support for assessing research impact activities

Support for impact metrics, such as impact factors and h indexes, was provided by 86% of libraries, with only 10% reporting that they had no plans to add this service (Figure 5). More than 50% of libraries supported altmetrics and online profile management, with close to 20% more planning to add these services.

## DISCUSSION

The findings from this study suggest that library research support aligns best with the planning, disseminating, and assessing impact phases of the research lifecycle. Data collected from biomedical researchers and library leaders confirm that library support is stronger at the beginning and end of the research lifecycle. However, a key distinction in the data collected from researchers suggests that their primary reliance on library support may be to use library resources rather than work with a librarian to achieve their aims.

The co-occurrence of coded data collected from researchers illustrates how library support is connected with almost all aspects of biomedical research, but its proximity to other clusters suggests that library support is not integrated throughout the workflows in the research lifecycle. Concerning for libraries, their support clusters with activities that the interviewed researchers did not explicitly state as needs, and the researchers did not include the traditional services of literature searches and systematic reviews. For example, all fifty-one library leaders who responded to the survey stated that they provided literature search services to researchers, with forty-seven providing systematic review services, but most interviewed researchers described conducting literature searches themselves.

Despite findings from this study suggesting that library support of the conducting phase of research is weaker than the other phases of research, evidence from the library leader survey suggests that many libraries are beginning to adapt their services. For instance, more than 25% of libraries have or plan to add support for data services, including data documentation, data wrangling, data collection, statistical methods, and data privacy. At the same time, a philosophical rift among health sciences libraries may be occurring, with 25% of libraries stating they had no plans to add these services.

### Implications of findings

Despite the discrepancies between researcher use of library support and services identified by library leaders, libraries appear to be well positioned to integrate their support within the research lifecycle. Findings from this study seek to build on the rich history of health sciences libraries developing research support services. These services include bioinformatics, research informationists, data management, and research evaluation support [12–16]. This study appears to confirm the finding by Vaughan et al. that researchers are unaware of scope of library services that are available to them [2]. Thus, libraries may need to increase their attention to marketing, relationship building, and collaboration.

### Transferability of findings

When transferring the findings from this study, library leaders should examine the research enterprise at their institutions and consider whether they can identify support gaps that are appropriate for their organizations. Research lifecycle models, such as those developed by University of Central Florida Libraries and Vaughan et al., can provide a framework for the types of activities to consider [1, 2]. It might also be prudent for library leaders to consider how they can mirror the strategic vision for NLM in becoming the epicenter of biomedical data at their institutions [3]. Regardless, the most important factor for libraries in considering their research support is institutional need.

When it comes to library support of the research lifecycle, there is no one best model, and not all library leaders agreed on the best course for the profession. When asked what additional steps libraries should be taking to meet the changing needs of biomedical research, one director responded, “This question assumes that health sciences libraries should be ‘taking steps’ to meet these ‘changing needs of biomedical research’. I’m not sure I agree with the premise.” She argued that librarians need more training in metadata, file-naming conventions, and how research happens if librarians are to provide meaningful support. Contrasting this perspective, another director stated, “All too often I see health sciences librarians locked into a mindset of being really good at something that does not need to be done or will soon reach its inevitable sunset (read systematic reviews). I believe that librarians need to hold true to their mission of knowledge support but have the ability to evolve along whatever path that will take us.”

### Limitations

Since the participants were allowed to deviate from the semi-structured interview protocol and because of constraints on participants’ time, not all concepts were fully explored. Low participation rates among researcher categories prevented an even distribution and limited comparison among categories. The quantitative survey instrument administered to library leaders used similar, but not exact, terminology in the qualitative interviews. It was possible that the interpretation of terms might differ between biomedical researchers and library leaders, inadvertently creating bias in the responses that were collected. Also, data collection, analysis, and interpretation of findings relied on the principal investigator of this study.

## CONCLUSION

Biomedical researchers described using the library primarily by accessing its collections. As might be expected, researchers’ most frequent use of assistance from librarians was in the form of literature searching and systematic reviews. However, in aggregate, researchers expressed comfort in searching online databases and accessing journal content without the need for assistance.

Data collected from library leaders suggest that libraries are heavily invested in traditional services supporting information retrieval. Findings from this study suggest that library services align with activities that occur at the beginning and end of the research lifecycle but not with the conducting phase of research. Future research on the research lifecycle might help increase libraries’ understanding of their role in biomedical research and position them as partners in accelerating the creation of new knowledge.

## SUPPLEMENTAL FILES

Appendix A Research lifecycles

Appendix B Potential sites for data collection

Appendix C Biomedical researcher interview instrument

Appendix D Health sciences library leadership survey instrument

Appendix E Description of categories and code definitions for biomedical researcher interviews

Appendix F Frequency of researcher interview codes (n=17)
