# Supplementary material for: Alignment of library services with the research lifecycle
Source: J Med Libr Assoc. 2019 Jul 1;107(3):384–93. doi: 10.5195/jmla.2019.595 (PMC6579601; doi:10.5195/jmla.2019.595)
Supplement: Appendix A [file jmla-107-384-s001.pdf]

JMLA Journal of the Medical Library Association

Supplemental content to
J Med Libr Assoc. Jul;107(3):dx.doi.org/10.5195/jmla.2019.595
www.jmla.mlanet.org
© Ragon 2019

Alignment of library services with the research lifecycle

Bart Ragon

APPENDIX A

Research lifecycles

Andrew Treloar: Diagrams, http://andrew.treloar.net/research/diagrams/

Bethel University: Preparing and Sharing Bethel Faculty Research Throughout the Research Lifecycle, https://blogs.bethel.edu/bethel-library/2014/08/28/preparing-and-sharing-bethel-faculty-research-throughout-the-research-lifecycle/

Bournemouth University: Research Lifecycle, http://blogs.bournemouth.ac.uk/research/research-lifecycle/

Cameron Neylon, Curtin University: From research life cycle to networks: The role of the library, http://www.slideshare.net/CameronNeylon/from-research-life-cycle-to-networks-the-role-of-the-library

Carol Hunter and Heather Gendron, University of North Carolina: Strategic Directions Along an Assessment Continuum, http://slideplayer.com/slide/2678735/

Center for Open Science: OSF, https://cos.io/osf/?gclid=CIqmtMnl4sYCFQqQaQodL-MB-Q

Elsevier, Library Connect: Q&A | Fostering research community through library spaces and services at The Ohio State University Research Commons, https://libraryconnect.elsevier.com/articles/qa-fostering-research-community-through-library-spaces-and-services-ohio-state-university

eResearch – Infrastructure and Communication (eRIC): Services supporting the research lifecycle, http://www.eric-project.org/services

Jill Sexton, University of North Carolina: Research HUB, http://slideplayer.com/slide/6891124/

London School of Hygiene and Tropical Medicine: Keep your Research Secure and address Open Access obligations, http://www.slideshare.net/lshtm/keep-your-research-secure-and-address-open-access-obligations

Mara Bordignon, York Library: Library Resources, http://slideplayer.com/slide/6851703/

Marieke Guy, University of Northhampton: Managing data throughout the research lifecycle, http://www.slideshare.net/MariekeGuy/research-lifecyclenorthampton

McGill University: E-Science Institute @ McGill, https://blogs.library.mcgill.ca/escienceinst/

Moira Bent, University of Newcastle, and Wendy White, University of Southhampton: Information in the research lifecycle, http://www.slideshare.net/guest8ec5ab/information-in-the-research-lifecycle

New York City College of Technology: Resources for Academic Publishing, http://libguides.citytech.cuny.edu/mcfp/finding

OpenWetWare: Headquarters/Research Pathway, http://openwetware.org/wiki/OpenWetWare:Headquarters/Research_Pathway

Simon Fraser University: Research Commons: Research lifecycle for graduate researchers, http://www.lib.sfu.ca/about/branches-depts/rc/research/research-lifecycle

University College London: Social media and research workflow, http://ciber-research.eu/download/20101111-social-media-report.pdf

JMLA Journal of the Medical Library Association

Supplemental content to
J Med Libr Assoc. Jul;107(3):dx.doi.org/10.5195/jmla.2019.595
www.jmla.mlanet.org
© Ragon 2019

University of British Columbia: Life Cycle of Scholarly Research, http://blogs.ubc.ca/pkp2009/files/2009/07/scholarly-research-cycle1.jpg

University of California San Francisco (UCSF): Questions About Clinical Research? UCSF's New HUB Website Has Answers, https://www.ucsf.edu/news/2012/03/11623/questions-about-clinical-research-ucsfs-new-hub-website-has-answers

University of Central Florida: Overview: Research Lifecycle, https://library.ucf.edu/about/departments/scholarly-communication/overview-research-lifecycle/

University of Illinois: Resources for Research, https://research.illinois.edu/resources-research

University of Melbourne: Research lifecycle, http://library.unimelb.edu.au/research/research-lifecycle

University of Nevada, Las Vegas: Library Support for the Research Lifecycle, https://www.youtube.com/watch?v=_gyWeutVEEk

University of Northhampton: Open access and the research lifecycle: a guide for researchers, https://researchsupporthub.northampton.ac.uk/2015/04/23/open-access-and-the-research-lifecycle-a-guide-for-researchers/

University of Oxford: Digital Services to Support Research Workshop, http://damaro.oucs.ox.ac.uk/dssr_workshop.xml

University of Sheffield: Staff Collaboration, https://www.sheffield.ac.uk/staff-collaboration/research-lifecycle/lifecycle

University of Victoria: The Library and the Research Cycle, http://libguides.uvic.ca/c.php?g=256706&p=2364848

University of Virginia Library: Steps in the Data Life Cycle, https://data.library.virginia.edu/data-management/lifecycle/

University of Western Australia: Research Data Management Toolkit: Research Lifecycle, http://guides.library.uwa.edu.au/c.php?g=325196&p=2178558

University of York: Information for researchers, https://www.york.ac.uk/library/info-for/researchers/

Wikimedia Commons: File:Research cycle.png, https://commons.wikimedia.org/wiki/File:Research_cycle.png
