# Supplementary material for: Alignment of library services with the research lifecycle
Source: J Med Libr Assoc. 2019 Jul 1;107(3):384–93. doi: 10.5195/jmla.2019.595 (PMC6579601; doi:10.5195/jmla.2019.595)
Supplement: Appendix B [file jmla-107-384-s002.pdf]

JMLA Journal of the Medical Library Association

SC

Supplemental content to
J Med Libr Assoc. Jul;107(3):dx.doi.org/10.5195/jmla.2019.595
www.jmla.mlanet.org
© Ragon 2019

Alignment of library services with the research lifecycle

Bart Ragon

APPENDIX B

Potential sites for data collection

1. Albany Medical College
2. Albert Einstein College of Medicine
3. Boston University Medical Center
4. Brown University, Alpert Medical School
5. Case Western Reserve University, School of Medicine
6. Charles R. Drew University of Medicine and Science
7. Cleveland Clinic
8. Columbia University
9. Creighton University
10. Dartmouth College
11. Drexel University Libraries
12. Duke University
13. East Carolina University
14. Eastern Virginia Medical School
15. Emory University
16. Florida International University
17. Florida State University
18. George Washington University Medical Center
19. Georgetown University Medical Center
20. Harvard Medical School
21. Howard University
22. Indiana University, School of Medicine
23. Johns Hopkins University
24. Loma Linda University
25. Louisiana State University Health Sciences Center, Shreveport
26. Louisiana State University, New Orleans
27. Loyola University Stritch School of Medicine
28. Mayo Clinic
29. Medical Coll of Wisconsin
30. Medical University of South Carolina
31. Meharry Medical College
32. Mercer University
33. Michigan State University
34. Miller School of Medicine, University of Miami
35. Morehouse School of Medicine
36. New York Medical College
37. New York University
38. Northwestern University, Feinberg School of Medicine
39. Ohio State University
40. Oregon Health & Science University
41. Penn State Hershey, Milton S. Hershey Medical Center
42. Rosalind Franklin University of Medicine and Science
43. Rowan University
44. Rush University Medical Center
45. Saint Louis University
46. Southern Illinois University
47. Stanford University Medical Center
48. Stony Brook University
49. Temple University
50. Texas A&M University
51. Texas Tech University Health Sciences Center, El Paso
52. Thomas Jefferson University
53. Tufts University
54. Tulane University
55. University at Buffalo, SUNY
56. University of Alabama, Birmingham

Supplemental content to
J Med Libr Assoc. Jul;107(3):dx.doi.org/10.5195/jmla.2019.595
www.jmla.mlanet.org
© Ragon 2019

57. University of Arizona
58. University of Arkansas for Medical Sciences
59. University of California, Davis
60. University of California, Los Angeles
61. University of California, Riverside
62. University of California, San Francisco
63. University of Central Florida, College of Medicine
64. University of Chicago
65. University of Cincinnati
66. University of Colorado, Anschutz Medical Campus
67. University of Connecticut Health
68. University of Florida
69. University of Hawaii, Manoa
70. University of Illinois, Chicago
71. University of Iowa
72. University of Kansas Medical Center
73. University of Kentucky
74. University of Louisville
75. University of Maryland, Baltimore
76. University of Massachusetts Medical Center
77. University of Michigan
78. University of Minnesota, Twin Cities
79. University of Mississippi Medical Center
80. University of Missouri
81. University of Missouri, Kansas City
82. University of Nebraska Medical Center
83. University of Nevada, School of Medicine
84. University of New Mexico
85. University of North Carolina, Chapel Hill
86. University of North Dakota
87. University of Oklahoma
88. University of Pennsylvania
89. University of Pittsburgh
90. University of Puerto Rico, Medical Sciences Campus
91. University of Rochester Medical Center
92. University of South Alabama
93. University of South Carolina
94. University of South Dakota, School of Medicine
95. University of South Florida
96. University of Southern California
97. University of Tennessee, Graduate School of Medicine
98. University of Texas Health Science Center
99. University of Texas Medical Branch
100. University of Texas Medical School, Houston
101. University of Texas Southwestern Medical Center
102. University of Texas, Rio Grande Valley
103. University of Toledo
104. University of Utah
105. University of Virginia
106. University of Washington
107. University of Wisconsin, Madison
108. Virginia Commonwealth University
109. Washington University, School of Medicine
110. Wayne State
111. Weill Cornell Medicine
112. Yale University
