# Supplementary material for: Alignment of library services with the research lifecycle
Source: J Med Libr Assoc. 2019 Jul 1;107(3):384–93. doi: 10.5195/jmla.2019.595 (PMC6579601; doi:10.5195/jmla.2019.595)
Supplement: Appendix C [file jmla-107-384-s003.pdf]

JMLA Journal of the Medical Library Association

SC
Supplemental content to
J Med Libr Assoc. Jul;107(3):dx.doi.org/10.5195/jmla.2019.595
www.jmla.mlanet.org
© Ragon 2019

Alignment of library services with the research lifecycle

Bart Ragon

APPENDIX C

Biomedical researcher interview instrument

Thank you for taking the time to meet with me today for this interview. Before we begin, I would like to review the procedure. The interview is designed to last sixty minutes but may take shorter depending on your responses. You have read and signed the consent agreement. Do you have any questions about the agreement?

I am interested in learning about the workflow of a biomedical researcher from identification of the research question to dissemination of knowledge. We will explore standard and emerging scientific practices, as well as the role of the library in supporting biomedical research. If you do not feel like answering a particular question or would like to stop the interview at any point, please let me know. The information that you give in the study will be handled confidentially. You have the right to withdraw from the study at any time without penalty.

Do you have any questions before we begin? May I start the audio recording?

Participant ID: ________________________________

Date: ________________________________

Introductory questions

1. Tell me a little about yourself. How did you get involved with biomedical research?
2. How long have you been conducting biomedical research?
3. Can you briefly describe the focus of your research area?
4. In your opinion, what are the most significant factors influencing biomedical science today?

Research workflow questions

Planning research

1. Prior to starting data collection, analysis, and interpretation, can you describe to me the major activities required to generate an idea and obtain funding?
    a. Probes: Background information (literature searching, systematic reviews, grey literature), citation management, locating data sources, bioinformatics tools (Blast, OncoMine, dbSNP, others), seeking grant funding, grant planning (writing, budget, application), methodology (experimental design, survey design, etc.), data management plans, data literacy, Biosketch creation, institutional review board (IRB) and institutional animal care and use committee (IACUC) protocols, ethics and compliance, identify collaborators, project planning, organizing and storing information.
    b. What are standard practices, what are emerging practices? Discuss education, consultation, and training needs of researcher or their team.

JMLA Journal of the Medical Library Association

SC
Supplemental content to
J Med Libr Assoc. Jul;107(3):dx.doi.org/10.5195/jmla.2019.595
www.jmla.mlanet.org
© Ragon 2019

2. Can you think of an instance where you used the library or asked for the help of a librarian to accomplish the activities in the research planning?
   a. Probes: Specific ways that they use or do not use library services based on the probes above. Use answers to guide conversation about why they use or do not use library services. Discuss how if the library had additional skills it may or may not demonstrate value. Are there other departments that provide support (who and how)?

Conducting research

1. Can you describe to me the major activities required to begin collecting and analyzing data?
   a. Probes: Automated and manual data collection, data documentation (file format, naming conventions, file organization), metadata standards, ontology/taxonomy, data security, versioning code (GitHub), versioning files, electronic lab notebook, data wrangling/cleaning (open source and/or proprietary software), data analysis and visualization (open source and/or proprietary software), statistics, geographic information system [GIS], research computing and technical support, scientific modeling, prototyping (maker technology), infrastructure and space, scheduling, project management, grant management.
   b. What are standard practices? What are emerging practices? Discuss education, consultation, and training needs of researcher or their team.
2. Can you think of an instance where you used the library or asked for the help of a librarian to accomplish the activities in the conducting research?
   a. Probe for specific ways that they use or do not use library services. Are there other departments that provide support (who and how)? Discuss education and training needs of researcher or their team. Use answers to guide conversation about why they use or do not use library services. Discuss how if the library had additional skills it may or may not be useful.

Disseminating research

1. Can you describe to me the major activities required to disseminate results?
   a. Probes: Journal selection for publication (for-profit publisher, open access), conference selection, web and social media marketing, bibliographic styles, writing center, preprint archive, author rights and copyright, image and graphics for submission, presentation poster preparation, data archiving, data sharing, long-term preservation of experiment materials (curation), institutional repository, funder public access policy compliance, grant citation.
   b. What are standard practices, what are emerging practices? Discuss education, consultation, and training needs of the researcher or their team.
2. Can you think of an instance where you used the library or asked for the help of a librarian to accomplish the activities in the disseminating research results?
   a. Probe for specific ways that they use or do not use library services. Are there other departments that provide support (who and how)? Discuss education and training needs or researcher or their team. Use answers to guide conversation about why they use or do not use library services. Discuss how if the library had additional skills it may or may not be useful.

JMLA Journal of the Medical Library Association

SC
Supplemental content to
J Med Libr Assoc. Jul;107(3):dx.doi.org/10.5195/jmla.2019.595
www.jmla.mlanet.org
© Ragon 2019

Assessing research impact

1. Can you describe to me the major activities required to measure the impact of the research?
    a. Probes: Citation metrics (h index, impact factor, times cited, others?), altmetrics and social media, managing research profile.
    b. What are standard practices? What are emerging practices? Discuss education, consultation, and training needs of researcher or their team.
2. Can you think of an instance where you used the library or asked for the help of a librarian to accomplish the activities in measuring the impact of research?
    a. Probe for specific ways that they use or do not use library services. Are there other departments that provide support (who and how)? Discuss education and training needs or researcher or their team. Use answers to guide conversation about why they use or do not use library services. Discuss how if the library had additional skills it may or may not be useful.
