# Supplementary material for: Alignment of library services with the research lifecycle
Source: J Med Libr Assoc. 2019 Jul 1;107(3):384–93. doi: 10.5195/jmla.2019.595 (PMC6579601; doi:10.5195/jmla.2019.595)
Supplement: Appendix D [file jmla-107-384-s004.pdf]

JMLA Journal of the Medical Library Association

SC

Supplemental content to
J Med Libr Assoc. Jul;107(3):dx.doi.org/10.5195/jmla.2019.595
www.jmla.mlanet.org
© Ragon 2019

Alignment of library services with the research lifecycle

Bart Ragon

APPENDIX D

Health sciences library leadership survey instrument

The purpose of the study is to investigate how health sciences libraries can expand their role in supporting the creation of knowledge within the research lifecycle. We are asking for one response per health sciences library from the director, deputy director, associate director, or comparable leadership level. Completing the questionnaire will take approximately twenty-five minutes. To assure confidentiality, I will not refer to your institution or your name in the data analysis and final report. You are heavily encouraged to devote at least ten minutes to the open-ended questions at the end of the survey.

Introductory questions

1. Name:
2. Institution:

3. How many years have you served at your current institution?

4. What is your work title?
   a. Director
   b. Deputy director
   c. Associate director
   d. Other (Please specify)

5. Skip logic based on the response for question 4.
   a. How many years in total have you served as a director, including multiple institutions?
   b. How many years in total have you served as a deputy director, including multiple institutions?
   c. How many years in total have you served as an associate director, including multiple institutions?
   d. How many years in total have you served at this position level, including multiple institutions?

JMLA Journal of the Medical Library Association

Supplemental content to
J Med Libr Assoc. Jul;107(3):dx.doi.org/10.5195/jmla.2019.595
www.jmla.mlanet.org
© Ragon 2019

Please indicate below any library services that you provide or plan to add supporting biomedical researchers?

	**Have provided more than 4 years**	**Have provided more than 2 years**	**Added in last 2 years**	**Plan to add in the next 2 years**	**Plan to add in the next 4 years**	**No plans to add**
Background literature searching						
Systematic reviews						
Grey literature searching						
Citation management						
Locating data sources						
National Center for Biotechnology Information (NCBI) tools (BLAST, GenBank, dbSNP, etc.)						
Seeking grant funding						
Writing center services						
Grant budget preparation						
Methodology						
Experimental design						
Data management plans						
Managing research data						
Data catalog						
Data literacy						
Biosketch creation						
Institutional review board (IRB) protocols						
Institutional animal care and use committee (IACUC) protocols						
Ethics and compliance						
Identifying collaborators						
Project planning and management						

JMLA Journal of the Medical Library Association

SC
Supplemental content to
J Med Libr Assoc. Jul;107(3):dx.doi.org/10.5195/jmla.2019.595
www.jmla.mlanet.org
© Ragon 2019

	**Have provided more than 4 years**	**Have provided more than 2 years**	**Added in last 2 years**	**Plan to add in the next 2 years**	**Plan to add in the next 4 years**	**No plans to add**
Methods for organizing and storing information						
Automated and manual data collection						
Data documentation (file format, naming conventions, file organization)						
Metadata standards						
Ontology/taxonomy						
Data privacy and security guidance						
Code versioning (GitHub, etc.)						
File versioning						
Electronic lab notebooks						
Data wrangling/ cleaning using open source tools such as R, Python, OpenRefine						
Data wrangling/ cleaning using proprietary software such as SAS, SPSS, Excel						
Data analysis and visualization using open source tools such as R or Python						
Data analysis and visualization using proprietary software such as SAS, SPSS, Excel						
Statistical methods						
Geographic information system (GIS)						
High performance computing (HPC)						
Scientific modeling						

JMLA Journal of the Medical Library Association

Supplemental content to
J Med Libr Assoc. Jul;107(3):dx.doi.org/10.5195/jmla.2019.595
www.jmla.mlanet.org
© Ragon 2019

	**Have provided more than 4 years**	**Have provided more than 2 years**	**Added in last 2 years**	**Plan to add in the next 2 years**	**Plan to add in the next 4 years**	**No plans to add**
Prototyping (3D printers and maker technology)						
Infrastructure and space						
Journal selection for publication						
Open access						
Conference selection						
Web and social media marketing						
Bibliographic styles						
Preprint archive						
Author rights and copyright						
Image and graphics for submission						
Presentation poster preparation						
Data archiving						
Data sharing						
Long-term preservation of experiment materials						
Institutional repository						
Funder public access policy compliance						
Grant citation						
Citation metrics (h index, impact factor, times cited)						
Altmetrics						
Online research profile management						

Please list examples of other actions (not previously mentioned in the survey) that your library has taken to accommodate the changing needs of biomedical research.

JMLA Journal of the Medical Library Association

SC
Supplemental content to
J Med Libr Assoc. Jul;107(3):dx.doi.org/10.5195/jmla.2019.595
www.jmla.mlanet.org
© Ragon 2019

Based on your experience, what new skills should health sciences libraries cultivate in order to more fully support biomedical research?

What additional steps should health sciences libraries be taking in order to meet the changing needs of biomedical research?

Over the course of your career, how has the role of health sciences libraries changed when it comes to supporting biomedical research?
