# Supplementary material for: Alignment of library services with the research lifecycle
Source: J Med Libr Assoc. 2019 Jul 1;107(3):384–93. doi: 10.5195/jmla.2019.595 (PMC6579601; doi:10.5195/jmla.2019.595)
Supplement: Appendix E [file jmla-107-384-s005.pdf]

JMLA Journal of the Medical Library Association

SC

Supplemental content to
J Med Libr Assoc. Jul;107(3):dx.doi.org/10.5195/jmla.2019.595
www.jmla.mlanet.org
© Ragon 2019

Alignment of library services with the research lifecycle

Bart Ragon

APPENDIX E

Description of categories and code definitions for biomedical researcher interviews

General

Competition: Concern for research ideas, funding, or preliminary research results to be obtained, discovered, or disseminated by another researcher.

Emerging practice: A practice in science that is changing due to evolutions in technology, culture, or external forces.

Influencing science: External forces affecting how researchers obtain funding, conduct science, or disseminate findings.

Library support: Support to the researcher or research teams provided by the library.

Licensing and venture: The process of transferring university intellectual property to a third party for the development of products, services, or applications. Also known as technology transfer.

Mentor/mentee: The role and relationship of mentors and mentees in the research lifecycle.

Nonlibrary support: Support to the researcher or research teams provided by nonlibrary personnel.

Reproducibility and replicability: Activities related to producing science in a way that another researcher can reproduce results of an experiment using the same data or to replicate the study with a different sample.

Planning research

Bioinformatics tools: National Center for Biotechnology Information (NCBI) tools or other forms of data such as Blast, OncoMine, dbSNP, etc.

Biosketch: Creation or management of biosketch information for use in grant applications.

Citation management: Methods used to store and reuse scholarly literature. Incudes software such as Endnote, Mendeley, apps, and other online tools.

Data literacy: Understanding of how to read and use data, the credibility of the source, and permissions and restrictions to its use.

Grant funding: The process of seeking grant funding, grant administration, or grant citation.

Grey literature: Seeking non-peer-reviewed information. Examples include conference proceedings, white papers, device manufacturing information, blog, wikis, etc.

Identify collaborators: The method in which collaborators are identified, includes the use of online tools or in-person networking opportunities.

IRB/IACUC: Activities related to obtaining or complying with the institutional review board (IRB) or the institutional animal care and use committee (IACUC).

Literature searching: Searching peer-reviewed literature databases such as PubMed, Google Scholar, etc.

Methodology: Experimental design and methods used to conduct a scientific experiment.

Systematic review: Comprehensive review of relevant literature, using a methodology, to provide a complete and exhaustive summary of the current literature relevant to a research question.

JMLA Journal of the Medical Library Association

Supplemental content to
J Med Libr Assoc. Jul;107(3):dx.doi.org/10.5195/jmla.2019.595
www.jmla.mlanet.org
© Ragon 2019

Conducting research

Collaborating: Activities of a researcher working with other researchers or those who support research.

Data analysis: The methods, techniques, and products employed to use and analyze data. Includes activities such as data wrangling or munging and data manipulation.

Data collection: Process of gathering data in research for the purpose of analysis.

Data management: The way that data are planned to be managed or are managed during the research process, either individually or as a team. Includes data documentation.

Data privacy and security: Activities associated with ensuring the protection of data, confidentiality, and compliance with applicable laws or policies.

Ethics: Knowledge of and adherence to policies and procedures required to conduct ethical research.

Lab notebook: Electronic or paper-based system for documenting activities connected to research.

Metadata: Use of data about data including metadata standards, ontologies, or taxonomies.

Open source software: Use of software in research that is open for further development and is maintained by a community of users.

Organizing and storing information: The methods that a researcher or research team organizes and stores electronic or non-electronic files, samples, or other objects.

Pilot experiment: Execution of an experiment prior to or within another experiment for the purposes of investigating potential future research.

Project management: Activities related to how a researcher or research team plans and manages the research process.

Proprietary software: Use of software in research owned by an individual or company.

Statistical methods: Techniques used in research to analyze and interpret data.

Disseminating research

Attending conferences: Value expressed by the researcher by attending a professional conference.

Author rights and copyright: Concepts related to the use of intellectual property of others or the rights transferred by researchers when publishing academic manuscripts.

Citation styles: Information necessary to cite a reference in a particular format used for publications or other scholarly reference.

Compliance: Adherence to policies and procedures associated with use of grant funding. Includes National Institutes of Health (NIH) public access policy compliance, submission of peer-reviewed articles into PubMed Central as required by NIH.

Conference selection: The process a researcher uses for selecting an academic conference to submit a presentation to.

Data preservation: The act of archiving and managing data post-research to ensure the data can continue to be accessed for as long as deemed necessary.

Journal selection: The process a researcher uses for selecting a peer-reviewed journal to submit an article for publication.

Open access: Access to, use of, or depositing of scholarly material connected to research. Includes open access articles, open access data, open access publishers, and open access repositories.

Preprint: Version of a publication made available prior to its publication in a scholarly journal.

JMLA Journal of the Medical Library Association

SC
Supplemental content to
J Med Libr Assoc. Jul;107(3):dx.doi.org/10.5195/jmla.2019.595
www.jmla.mlanet.org
© Ragon 2019

Presentation: Activities related to presenting scholarly work include the creation of graphics and PowerPoint poster design.

Social media: Use of social media to promote science, awareness, or reputation management.

Writing: Process or activities related to writing journal papers and grants.

Assessing research impact

Altmetrics: Collection and/or use of nontraditional metrics of scholarly work, including articles, data, and other products of research. Can include downloads, views, or mentions in social media.

Citation metrics: Collection and/or use of traditional metrics connected to scholarly publications of a researcher's work. Metrics include number of citations, h index, impact factor, etc.
