# Supplementary material for: Alignment of library services with the research lifecycle
Source: J Med Libr Assoc. 2019 Jul 1;107(3):384–93. doi: 10.5195/jmla.2019.595 (PMC6579601; doi:10.5195/jmla.2019.595)
Supplement: Appendix F [file jmla-107-384-s006.pdf]

JMLA Journal of the Medical Library Association

Supplemental content to
J Med Libr Assoc. Jul;107(3):dx.doi.org/10.5195/jmla.2019.595
www.jmla.mlanet.org
© Ragon 2019

Alignment of library services with the research lifecycle

Bart Ragon

APPENDIX F

Frequency of researcher interview codes (n=17)

Conceptual categories	Code	# of references
General	Mentor/mentee	73
	Nonlibrary support	50
	Emerging practice	47
	Influencing science	44
	Library support	39
	Reproducibility and replicability	14
	Competition	8
	Licensing and venture	5
Planning research	Grant funding	66
	Literature searching	40
	Methodology	35
	Identify collaborators	30
	Grey literature	17
	Citation management	17
	Data literacy	14
	Institutional review board (IRB)/institutional animal care and use committee (IACUC)	13
	Biosketch	12
	Systematic review	10
	Bioinformatics tools	8
Conducting research	Data analysis	59
	Collaborating	57
	Data collection	47
	Data management	41
	Open source software	35
	Organizing and storing information	34
	Proprietary software	26
	Data privacy and security	18
	Project management	18
	Lab notebook	17

SC
Supplemental content to
J Med Libr Assoc. Jul;107(3):dx.doi.org/10.5195/jmla.2019.595
www.jmla.mlanet.org
© Ragon 2019

Conceptual categories	Code	# of references
	Statistical methods	17
	Pilot experiment	13
	Metadata	5
	Ethics	1
Disseminating research	Open access	53
	Writing	33
	Social media	32
	Journal selection	29
	Conferences attendance	21
	Data preservation	17
	Presentation	15
	Compliance	12
	Conference selection	11
	Author rights and copyright	4
	Preprint	3
	Citation styles	1
Assessing research impact	Citation metrics	30
	Altmetrics	5
